# Cyanin Chloride Inhibits Hyperbaric Pressure-Induced Decrease of Intracellular Glutamate-Aspartate Transporter in Rat Retinal Müller Cells

**DOI:** 10.1155/2018/6128470

**Published:** 2018-10-31

**Authors:** Xiaomin Chen, Yue Wang, Fangfang Han, Min Ke

**Affiliations:** Department of Ophthalmology, Zhongnan Hospital, Wuhan University, Wuhan, China

## Abstract

**Purpose:**

Glaucoma is the leading cause of irreversible blindness throughout the world. The pathogenesis of glaucoma is complex, and neuroprotection is a crucial aspect of therapy. High concentrations of extracellular glutamate are toxic to the optic nerve. The glutamate-aspartate transporter (GLAST) in retinal Müller cells is involved in the development of glaucoma. Anthocyanin has been reported to protect retinal neurons. We hypothesize that cyanin chloride, a type of anthocyanin, can inhibit hyperbaric pressure-induced GLAST decreases in cultured rat retinal Müller cells and may serve as a potential neuroprotective agent in glaucoma treatment.

**Materials and Methods:**

Sprague Dawley rat Müller cells were cultured in a hyperbaric pressure device at 60 mmHg additional pressure and treated with cyanin chloride (10 *μ*mol/L, 30 *μ*mol/L, or 50 *μ*mol/L) or vehicle for 2 hours. Cell survival rates (SRs) were evaluated by an MTT assay. GLAST mRNA and protein expression were determined by western blot and RT-PCR analyses, respectively.

**Results:**

Cell SR was significantly decreased in the 60 mmHg additional hyperbaric pressure group compared to the control group (*P* < 0.01). Cyanin chloride treatment significantly improved SR under 60 mmHg additional pressure (*P* < 0.01). GLAST mRNA and protein expression levels in Müller cells were significantly reduced in the 60 mmHg hyperbaric pressure group compared to the control group (*P* < 0.01), but cyanin chloride significantly inhibited hyperbaric pressure-induced decreases in GLAST expression (*P* < 0.01).

**Conclusion:**

Our results support our hypothesis and demonstrate that cyanin chloride can protect rat retinal Müller cells from hyperbaric pressure-induced decreases of GLAST.

## 1. Introduction

Glaucoma, characterized by the death of retinal ganglion cell neurons and subsequent visual dysfunction, is the leading cause of irreversible blindness worldwide [1]. The pathogenesis of glaucoma is complex and not fully elucidated. A series of pathological changes contribute to the development of the disease, including obstruction of retrograde transport of axial plasma flow, caused by high intraocular pressure; ischemia and reperfusion injury; oxidative stress; glutamate excitatory toxicity; abnormal immune response; and glial activation [2–7]. Clinically, glaucoma is primarily treated by reducing intraocular pressure (IOP). However, it is commonly known that both retinal ganglion cell (RGC) death and optic nerve damage can occur independently of IOP, and loss of RGCs can continue despite IOP reduction in some patients [8, 9]. Recently, neuroprotective approaches against excitotoxic glutamate have been investigated as potential therapy for optic neuropathies [10, 11].

Glutamate is one of the most important excitatory neurotransmitters in the mammalian central nervous system (CNS), including the retina [12]. However, its accumulation in extracellular spaces is excitotoxic to neurons through activation of glutamate receptors [13]. Glutamate excitotoxicity has been proposed to be an important contributor to the death of CNS neurons in conditions ranging from acute ischemic stroke to chronic neurodegenerative diseases such as Alzheimer's disease [14, 15]. In the eye, glutamate excitotoxicity has been implicated in RGC death in glaucoma and ischemia-related conditions such as diabetic retinopathy [16–21]. Researchers have detected excessive levels of glutamate in glaucoma [8, 22]. Dreyer et al. investigated elevated glutamate concentrations in the vitreous body of both humans and monkeys with glaucoma [22], and Brooks et al. showed that eyes from dogs with primary glaucoma also had high vitreal glutamate expression [8]. Furthermore, experiments support the idea that excessive glutamate induces RGC death both *in vivo* and *in vitro* [23–26]. However, the exact mechanism of glutamate-induced RGC death with elevated IOP remains to be elucidated. One of the leading hypotheses is that ocular hypertension causes glutamate transporter dysfunction, leading to the excessive glutamate increase in the extracellular space. This induces excessive increases in intracellular calcium-ion concentration or oxidative stress and leads to apoptosis [27–31].

In the retina, glutamate is metabolized via the glutamate-glutamine cycle between the neurons and glial cells. Müller cells, the principal retinal glial cells, play an important role in maintaining normal retina morphology and function, including supporting nerve cells in the retina, regulating the retinal environment, and transmitting and integrating retinal nerve signals [17, 32]. Glutamate transporters play a key role in the glutamate-glutamine cycle. To date, five excitatory amino acid transporters (EAAT1–5) have been identified that may be significant in the clearance of glutamate in the nervous system [33, 34]. In the retina, EAAT1, also referred to as GLAST, is found in Müller cells [34]. If excessive extracellular glutamate is implicated in neuronal loss, the possibility of a transporter abnormality should be considered. Some studies have shown decreased GLAST concentration both in human patients with glaucoma and in a rat model of glaucoma [27, 35]. Therefore, reduced GLAST function may contribute to the elevated glutamate found in the vitreous of patiens with glaucoma.

In contrast to the damaging effects of decreased GLAST, several lines of evidence have shown that anthocyanin can protect retinal neurons *in vivo* and *in vitro* [36, 37]. In our former study, cyanin chloride (a type of anthocyanin) improved GLAST expression in rat retinal Müller cells cultured in high glucose [38]. We hypothesize that cyanin chloride can protect against decreased GLAST activity and may serve as a potential neuroprotective agent in glaucoma treatment. We propose to test this hypothesis by culturing rat retinal Müller cells in a hyperbaric chamber, to simulate the effects of increased IOP.

## 2. Materials and Methods

### 2.1. Cell Culture

Müller cells were obtained from the College of Life Sciences, Wuhan University (Wuhan, China). The cells were cultured in Dulbecco's modified Eagle's medium (DMEM; Gibco Life Technologies, Carlsbad, CA, USA) supplemented with 100 U/ml penicillin, 100 *µ*g/ml streptomycin, and 10% fetal bovine serum (FBS; Gibco Life Technologies) in a 25 cm^2^ culture flask.

In our previous studies, different hyperbaric pressure levels (15 mmHg additional pressure/30 mmHg additional pressure/45 mmHg additional pressure/60 mmHg additional pressure) were used to test Müller cell survival and GLAST protein expression. Our data showed that 60 mmHg additional pressure exhibited the most pronounced effect on cell survival and GLAST protein levels, compared with the atmospheric pressure control [39].

The cells were cultured in five groups. When they reached 80–90% confluence, the cells were exposed to atmospheric or hyperbaric pressure for 2 h. Then, the cells exposed to hyperbaric pressure were treated with the different concentrations of cyanin chloride, and the cells were cultured for an additional 3 days: Group A (control group): 0 mmHg additional pressure without cyanin chloride treatment; Group B (hyperbaric group): 60 mmHg additional pressure; Group C: 60 mmHg additional pressure and 10 *μ*mol/L cyanin chloride; Group D: 60 mmHg additional pressure and 30 *μ*mol/L cyanin chloride; and Group E: 60 mmHg additional pressure and 50 *μ*mol/L cyanin chloride.

### 2.2. Pressure Device

A T25 culture flask (Corning, USA) was equipped with a manometer (Yueqing City Supreme Electric Co. Ltd., Shanghai, China) and placed in an incubator at 37°C. A mixture of 95% air and 5% CO_2_ was pumped into the chamber to obtain 60 mmHg pressure. The pressure was adjusted every 10 min for the 2-hour exposure period to maintain a constant pressure of 60 mmHg.

### 2.3. Cell Morphology

The cells were viewed under an Axio microscope (Zeiss, Oberkochen, Germany), and images were acquired with a digital camera (Canon, Tokyo, Japan).

### 2.4. MTT

The cells from each group were seeded in 96-well plates at a density of 5 × 10^5^ cells per well. After overnight culture, MTT (Sigma-Aldrich, USA) was added to each well at a concentration of 0.5 mg/ml and incubated for an additional 4 h. A 150 *μ*L aliquot of DMSO was added for 10 min to induce a reaction. The absorbance was measured at a wavelength of 570 nm in a microplate spectrophotometer (Multiskan MK3, Thermo Fisher Scientific, USA).

### 2.5. Real-Time PCR

RNA was extracted from Müller cells using the Trizol reagent (Invitrogen) according to the manufacturer's protocol and stored at −80°C. The concentration and purity of the RNA preparations were determined by measuring the absorbance at 260/280 nm. RNA was reverse transcribed into complementary DNA using a reverse transcription kit (Fermentas, Canada). SYBR Real-Time PCR Master Mix (Fermentas) was used to conduct real-time PCR analyses. The following primer pairs were used: 5′-GGGGAACTCCGTGATTGA-3′ (sense) and 5′-CATCTTGGTTTCGCTGTCT-3′ (antisense) for GLAST and 5′-CACGATGGAGGGGCCGGACTCATC-3′ (sense) and 5′-TAAAGACCTCTATGCCAACACAGT-3′ (antisense) for *β*-actin. The reactions were performed in triplicates. The comparative Ct (ΔΔCt) method was used to obtain quantitative data of relative gene expression according to the manufacturer's instructions. Relative GLAST expression levels were normalized to *β*-actin.

### 2.6. Western Blot Analysis

The cells were homogenized in the RIPA lysis buffer (Bigtime, China) containing a protease inhibitor cocktail (Beyotime, China) and PMSF (Bigtime, China). Proteins were separated on 12% SDS-PAGE gels (Sigma-Aldrich, St. Louis, MO, USA) and transferred to nitrocellulose filter membranes. Membranes were blocked in Tris-buffered saline containing 5% fat-free milk and incubated overnight at 4°C with antibodies against GLAST (1 :200; ab416, Abcam). The membranes were then incubated with horseradish peroxidase-linked secondary antibodies against rabbit IgG (1 : 5000; Invitrogen) for 1 h at room temperature in the dark. Bands were visualized by exposure to the Kodak X-ray film. Image analysis and densitometry were performed by ImageJ.

### 2.7. Statistical Analysis

Statistical analyses were performed using SPSS 19.0 statistical software (IBM SPSS, Chicago, IL, USA). One-way ANOVA was used to compare differences among three or more groups. *P* value <0.05 was considered statistically significant.

## 3. Results

**Figure 1:**
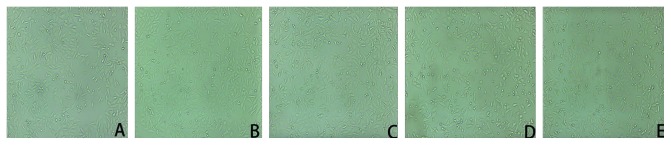
Cell morphology observed under inverted-phase microscopy (×100). Müller cells were cultured to 80–90% confluence and exposed to hyperbaric or atmospheric pressure for two hours and then to varying concentrations of cyanin chloride (or vehicle) for three days before images were captured. (A) Group A: 0 mmHg; (B) Group B: 60 mmHg; (C) Group C: 60 mmHg + 10 *μ*mol/L cyanin chloride; (D) Group D: 60 mmHg + 30 *μ*mol/L cyanin chloride; (E) Group E: 60 mmHg + 50 *μ*mol/L cyanin chloride.

Under the light microscope, normal Müller cell bodies appeared oblong, star shaped, spindled, and pyramidal (Figure 1). Cell morphology did not change significantly after exposure to either hyperbaric pressure (Figure 1) or cyanin chloride (Figures 1–1).

**Figure 2:**
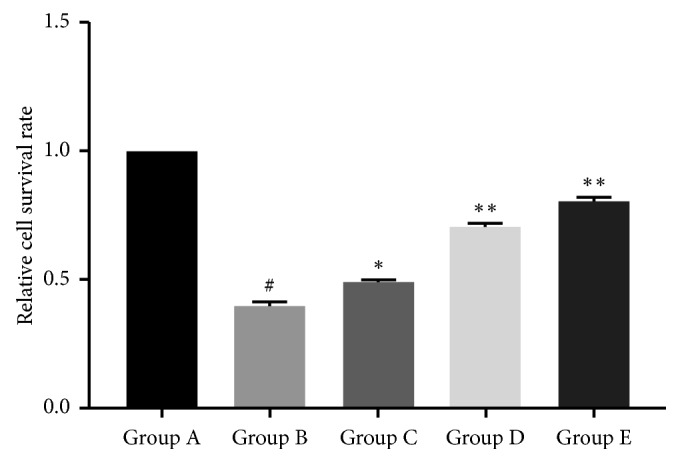
Relative cell survival rate in Müller cells. The cells were treated as described in Figure 1 and then incubated with MTT to determine the relative survival rate. ^*#*^*P* < 0.0001, compared with Group A (0 mmHg additional pressure); ^*∗*^*P* < 0.0005 and ^*∗∗*^*P* < 0.0001, compared with Group B (60 mmHg additional pressure). Data from one experiment are shown as a representative of three studies performed under the same conditions. Group A: 0 mmHg; Group B: 60 mmHg; Group C: 60 mmHg + 10 *μ*mol/L cyanin chloride; Group D: 60 mmHg + 30 *μ*mol/L cyanin chloride; Group E: 60 mmHg + 50 *μ*mol/L cyanin chloride.

The MTT assay was used to evaluate the cell survival rate (SR). The cell SRs of Groups A to E, respectively, were 1.2665 ± 0.0399, 0.5129 ± 0.0259, 0.6232 ± 0.0247, 0.8987 ± 0.0389, and 1.0293 ± 0.0421, and accordingly, the relative values were 100%, 40.33%, 49.33%, 71%, and 81%. The relative SR was significantly decreased in Group B (+60 mmHg additional pressure) compared to Group A (atmospheric pressure) (*P* < 0.01; Figure 2), but treatment with cyanin chloride increased survival in Groups C, D, and E compared to Group B, and the effect was dose-dependent (*P* < 0.01; Figure 2).

**Figure 3:**
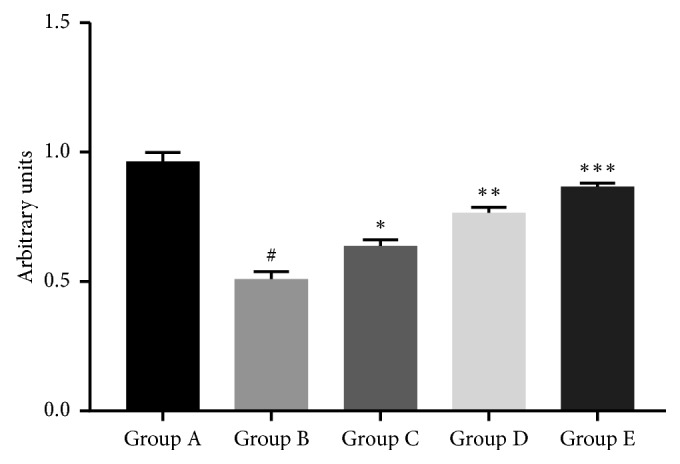
RT-PCR analysis of GLAST mRNA expression levels in retinal Müller cells. The cells were treated as described in Figure 1. ^*#*^*P* < 0.0001, compared with Group A (0 mmHg additional pressure); ^*∗*^*P* < 0.05, ^*∗∗*^*P* < 0.001, and ^*∗∗∗*^*P* < 0.0001, compared with Group B (60 mmHg additional pressure). Data from one experiment are shown as a representative of three studies performed under the same conditions. Group A: 0 mmHg; Group B: 60 mmHg; Group C: 60 mmHg + 10 *μ*mol/L cyanin chloride; Group D: 60 mmHg + 30 *μ*mol/L cyanin chloride; Group E: 60 mmHg + 50 *μ*mol/L cyanin chloride.

**Figure 4:**
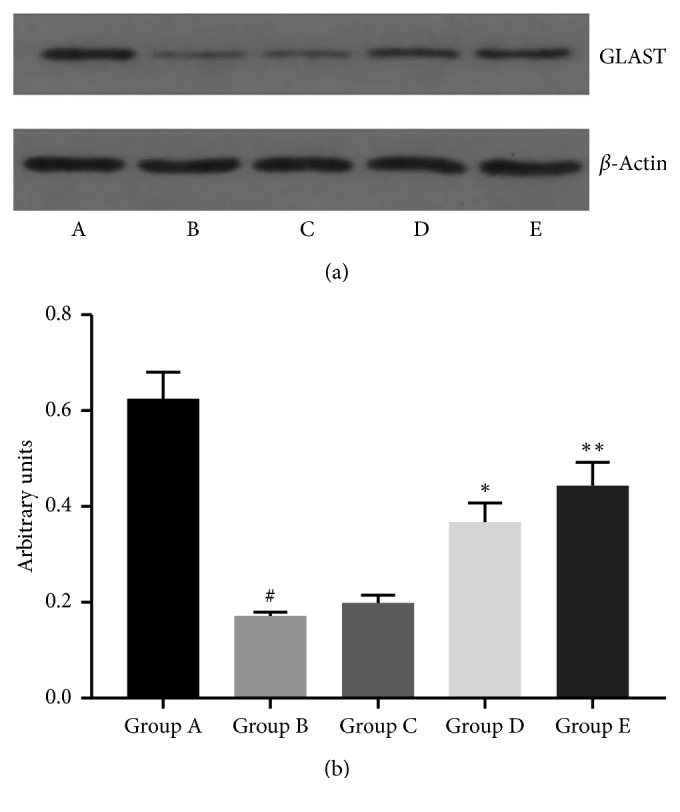
GLAST protein expression levels in retinal Müller cells. The cells were treated as described in Figure 1. (a) Representative western blot shows GLAST expression; (b) quantification of GLAST protein expression. ^*#*^*P* < 0.0001, compared with Group A (0 mmHg additional pressure); ^*∗*^*P* < 0.01 and ^*∗∗*^*P* < 0.001, compared with Group B (60 mmHg additional pressure). Data from one experiment are shown as a representative of three studies performed under the same conditions. Group A: 0 mmHg; Group B: 60 mmHg; Group C: 60 mmHg + 10 *μ*mol/L cyanin chloride; Group D: 60 mmHg + 30 *μ*mol/L cyanin chloride; Group E: 60 mmHg + 50 *μ*mol/L cyanin chloride.

We investigated the effect of cyanin chloride on GLAST mRNA and protein expression in cultured retinal Müller cells under hyperbaric pressure. The mRNA analysis revealed nearly a 50% reduction in GLAST mRNA expression in the cells exposed to 60 mmHg additional pressure (Group B), compared with the control (Group A). This effect was reversed in a dose-dependent manner in the treatment groups (Figure 3). Western blot analyses revealed a similar but not identical picture. GLAST protein expression was decreased by 70% in the cells exposed to hyperbaric pressure (Group B), compared to control (Group A) (*P* < 0.05; Figure 4). While GLAST protein expression was significantly elevated in Groups D and E (*P* < 0.05; Figure 4), 10 *μ*mol/L cyanin chloride did not rescue GLAST expression.

## 4. Discussion

Our results confirm our hypothesis that cyanin chloride, a type of anthocyanin, can improve the GLAST level in hyperbaric pressure-cultured rat retinal Müller cells. In the present study, we found that hyperbaric pressure inhibited the GLAST level and was accompanied with Müller cell death, and cyanin chloride reversed this effect. Our findings suggest that cyanin chloride may be a potential therapeutic agent for glaucoma.

Anthocyanins belong to the most common class of phenolic compounds, which is the group of water-soluble pigments [40]. These plant pigments that are widely distributed in berries, dark grapes, and other pigmented fruits and vegetables [40] and anthocyanins have numerous protective effects, including antioxidant, anti-inflammatory, antiproliferative, antimutagenic, antimicrobial, antiallergic, and anticarcinogenic effects; protect from cardiovascular damage; improve microcirculation; prevent diabetes; and improve vision [40–43].

In some observational and clinical trials, consumption of high amounts of fruits and vegetables rich in phenolics is associated with a reduced risk of major age-related eye diseases such as cataracts, glaucoma, and age-related macular degeneration (AMD) [44]. Anthocyanins can also help prevent diabetic retinopathy and retinitis pigmentosa [45]. It has been reported that anthocyanin exerts its retinoprotective effect by scavenging free radicals [46]. In addition, anthocyanins have been shown to reduce *N*-methyl-*N*-nitrosourea-induced retinal degeneration in rats [37]. Furthermore, oral administration of black currant anthocyanins induced a significant decrease in IOP levels in glaucoma patients [47].

In our study, 30 *µ*mol/L and 50 *µ*mol/L cyanin chloride increased GLAST protein levels in Müller cells exposed to hyperbaric pressure. GLAST is a sodium-dependent transporter that transports extracellular glutamate into Müller cells using free energy established by the Na^+^-K^+^-ATPase [48]. Since ATP production is reduced as hyperbaric pressure increases [49], the extracellular glutamate level increasing by Müller cells is reduced, leading to an increase in extracellular glutamate concentration. Consistent with our findings, it has been reported that hyperbaric pressure increases glutamate toxicity to RGCs [23]. Increased extracellular glutamate may be responsible for the decreased survival of Müller cells exposed to hyperbaric pressure. Increased GLAST levels may promote metabolism of extracellular glutamate into Müller cells, thereby preventing glutamate excitotoxicity. In addition, we found that hyperbaric pressure significantly decreased the GLAST level, suggesting that increased pressure may also lead to decreased metabolism capacity by the cells.

The mechanism by which anthocyanin increases the GLAST level under hyperbaric pressure has not yet been elucidated. GLAST expression can be regulated via DNA transcription, mRNA splicing and degradation, and protein synthesis and localization [23]. Oxygen-free radicals can oxidize the glutamate transporter proteins [50]. Increasing evidence suggests possible involvement of oxidative alterations to glutamate transporters in specific pathologies of excitotoxic neurodegeneration [51]. Thus, the antioxidant effect of anthocyanin may contribute to its role in GLAST regulation. Future studies are warranted to identify the mechanisms underlying GLAST regulation in Müller cells under hyperbaric pressure. The limitation of our study is that we did not test the effect of anthocyanin on Müller cells under atmospheric pressure condition. The reason is that we were trying to imitate the glaucomatic condition *in vivo* and were focused on observing the protective effect of the drug under high-pressure conditions. The biotinylation experiments and functional assays will be done to further investigate the transportation of flats [52].

In conclusion, we have demonstrated that cyanin chloride prevents hyperbaric pressure-induced decreases in survival of cultured Müller cells, and this effect is mirrored by the increased GLAST level. Our findings suggest that cyanin chloride may be a useful therapeutic approach in the management of glaucoma and other diseases mediated by chronic excitotoxicity.

## 5. Conclusion

The results support our hypothesis and demonstrate that cyanin chloride can protect rat retinal Müller cells from hyperbaric pressure-induced decreases in the GLAST level.

## Data Availability

The data used to support the findings of this study are included within the article.
